# Anaerobic Fungi: A Potential Source of Biological H_2_ in the Oceanic Crust

**DOI:** 10.3389/fmicb.2016.00674

**Published:** 2016-05-12

**Authors:** Magnus Ivarsson, Anna Schnürer, Stefan Bengtson, Anna Neubeck

**Affiliations:** ^1^Department of Palaeobiology and Nordic Center for Earth Evolution, Swedish Museum of Natural HistoryStockholm, Sweden; ^2^Department of Microbiology, Uppsala BioCenter, Swedish University of Agricultural SciencesUppsala, Sweden; ^3^Department of Geological Sciences, Stockholm UniversityStockholm, Sweden

**Keywords:** anaerobic fungi, deep biosphere, ocean crust, chemoautotrophs, fungal interactions

## Abstract

The recent recognition of fungi in the oceanic igneous crust challenges the understanding of this environment as being exclusively prokaryotic and forces reconsiderations of the ecology of the deep biosphere. Anoxic provinces in the igneous crust are abundant and increase with age and depth of the crust. The presence of anaerobic fungi in deep-sea sediments and on the seafloor introduces a type of organism with attributes of geobiological significance not previously accounted for. Anaerobic fungi are best known from the rumen of herbivores where they produce molecular hydrogen, which in turn stimulates the growth of methanogens. The symbiotic cooperation between anaerobic fungi and methanogens in the rumen enhance the metabolic rate and growth of both. Methanogens and other hydrogen-consuming anaerobic archaea are known from subseafloor basalt; however, the abiotic production of hydrogen is questioned to be sufficient to support such communities. Alternatively, biologically produced hydrogen could serve as a continuous source. Here, we propose anaerobic fungi as a source of bioavailable hydrogen in the oceanic crust, and a close interplay between anaerobic fungi and hydrogen-driven prokaryotes.

## Introduction

The oceanic igneous crust is one of the few great frontiers of unknown biology on Earth (Schrenk et al., 2009; Edwards et al., 2012), and despite it being the largest potential habitat for microbial life, next to nothing is known about the abundance, diversity, and ecology of its biosphere. With a few exceptions (Mason et al., 2010; Orcutt et al., 2010; Lever et al., 2013), our understanding of the biosphere of the subseafloor crust is based on a fossil record (Staudigel et al., 2008; Ivarsson et al., 2012). Unexpectedly, a considerable part of the fossilized microorganisms are now interpreted as remnants of fungi rather than filamentous prokaryotes (Schumann et al., 2004; Ivarsson et al., 2012, 2013; Bengtson et al., 2014).

The presence of fungi in deep subseafloor basalts raises several questions regarding their metabolism since all known fungal species are heterotrophs and thus require a steady supply of accessible carbohydrates to sustain long-term communities. Organic compounds accessible for fungal metabolism in the oceanic crust could be produced abiotically by fluid rock interactions (mostly compounds ranging from C_1_ to C_4_), but difficult to quantify at such depths. It is also questionable whether abiotic sources are able to ensure a steady supply of organic compounds over geological time. Ingress of organic matter by seawater could be another source but restricted to shallow parts of the oceanic crust (Ivarsson et al., 2015c, 2016). An alternative source could be biological organic compounds provided by lithoautotrophic communities (Bengtson et al., 2014; Ivarsson et al., 2015a).

Molecular hydrogen appears to be the most important energy source available to the deep-biosphere communities (Nealson et al., 2005). Studies suggest that the tree of life roots in non-photosynthetic hydrogen-driven communities, and such communities persist in the deep biosphere until this day (Nealson et al., 2005). Metabolic products from such communities, such as acetate, could serve as accessible carbon sources for fungi. But an even more important source for organic molecules should be the communities themselves. Fungi grow by decomposing various carbohydrates, thus a hydrogen-driven lithoautotrophic community could serve as carbon source for a fungal heterotrophic community. Molecular hydrogen has been assumed to be the result of abiogenic processes such as hydrous alteration of ultramafic rocks (serpentinization; Nealson et al., 2005; McCollom, 2007). Basalt-hosted systems in general produce much lower levels of molecular hydrogen compared to ultramafic-hosted systems (McCollom, 2007), and the abiotic production of molecular hydrogen is probably insufficient to ensure a steady supply and support a hydrogen-driven ecosystem over geological periods (Anderson et al., 1998). Biologically produced hydrogen has been proposed by Parkes et al. (2011) as an alternative source, and laboratory experiments showed that the production of molecular hydrogen in the presence of prokaryotes was higher than the abiotic production of molecular hydrogen.

Most fungi are aerobic, but anaerobic fungi have been found in freshwater lakes, landfill sites (McDonald et al., 2012), deep-sea sediments (Nagano and Nagahama, 2012), and rumens of herbivores (Khejornsart and Wanapat, 2010; Liggenstoffer et al., 2010). Moreover, they have been shown to be part of the microbial community in biogas reactors (Haitjema et al., 2014). These fungi have highly active polysaccharide-degrading enzymes, making them interesting for biomass degradation and different biotechnological applications (Kazda et al., 2014). Instead of mitochondria, anaerobic fungal species have hydrogenosomes, organelles that contain hydrogenase and produce molecular hydrogen, carbon dioxide, acetate, and other compounds as metabolic waste products (Yarlett et al., 1986; Brul and Stumm, 1994; Khejornsart and Wanapat, 2010; Gruninger et al., 2014). The biological production of molecular hydrogen in the rumen stimulates methanogenesis, and anaerobic fungi and methanogenic archaea exist in a vital relationship that increases the metabolic rate compared to the single-fungus system (Khejornsart and Wanapat, 2010; Gruninger et al., 2014). A similar co-operation would be likely to assume for other anoxic environments, like the oceanic crust.

Anoxic provinces are widespread in the deep-sea sediments but also in the underlying igneous crust. Anaerobic hydrogen-consuming prokaryotes such as methanogens have been reported from the igneous crust (Cowen et al., 2003; Orcutt et al., 2010; Lever et al., 2013), as have fungi (Ivarsson et al., 2012; Hirayama et al., 2015). Even though fungi so far have been neglected in most metagenomic studies, a few reports have shown the presence of anaerobic fungi in deep-sea sediments and on seafloor-exposed basalts (Damare et al., 2006; López-García et al., 2007; Nagano and Nagahama, 2012).

We suggest anaerobic fungi as a biological source of hydrogen in anoxic provinces in the igneous oceanic crust, and that they provide a base for hydrogen-consuming prokaryotes. In deep-drilled subseafloor basalts consortia of fungi and chemoautotrophic prokaryotes indicate a close symbiotic relationship between the eukaryotic and prokaryotic microorganisms, enabling colonization and establishment of vital communities (Bengtson et al., 2014; Ivarsson et al., 2015a). By degrading the organic matter (carbohydrates such as polysaccharides) from the prokaryotic cells, the fungi would be able to produce molecular hydrogen, carbon dioxide, and acetate, available as substrate for the chemoautotrophic community. Although anaerobic fungi have not yet been isolated from samples of the oceanic igneous crust, we suggest based on the presence of fossilized fungal-prokaryotic consortia and anaerobic fungi from seafloor exposed basalt, that anaerobic fungi and hydrogen-consuming prokaryotes exist together, interacting in similar ways as in such a different environment as the rumen.

## Oxygen in the Oceanic Crust

The distribution of dissolved oxygen in the igneous oceanic crust is poorly understood because of technical limitations in sampling and monitoring. Dissolved oxygen is introduced to the oceanic crust by seawater recharge at basaltic outcrops, and its further propagation through the system is controlled by the present fluid regime. Thus, a range of various parameters like sediment cover, permeability, porosity, and depth and age of the crust control the extension and longevity of dissolved oxygen. Besides, oxygen has the highest redox potential of all electron acceptors and is readily consumed by fluid–rock interactions and microbial activity (Bach and Edwards, 2003). The oxygen consumption rate in marine sediments, for instance, is reflected by the overall microbial activity, thus, in sediments with moderate to high content of organic matter, oxygen is consumed in the first few millimeters to centimeters, whereas in organic-poor sediments oxygen can persist for meters (Orcutt et al., 2011). Oxygen consumption in subseafloor basalts is poorly constrained, but circulations of oxic fluids occur through cool regions (Bach and Edwards, 2003). At North Pond, the western flank of the Mid-Atlantic Ridge, deep anoxic sediments are oxygenized owing to upflow of oxic fluids from the underlying igneous crust. Orcutt et al. (2013) further calculated oxygen consumption rates of 1 nmol cm^-3^_ROCK_ d^-1^ or less in the upper sections of the young (∼8 Ma), and cool (<25°C) basaltic crust at North Pond. However, ridge-flanks are areas where the igneous crust is exposed, and where much of the fluid exchange between the ocean and the basement is focused (Bekins et al., 2007; Fisher and Wheat, 2010). Anoxic conditions may prevail at least locally in off-axis areas covered by sediments, as well as in deep settings where the recharge is restricted or absent and most of the oxygen is being consumed (Bach and Edwards, 2003; Orcutt et al., 2011, 2013).

In a heterogeneous setting, such as the subseafloor basalt where fluid circulation is controlled by the interconnectedness of pore space, local conditions tend to vary greatly on a micro-scale. Even though oxygenated fluids may penetrate deep into basalt, local pockets of anoxic conditions occur frequently. Especially in massive basalts, circulation of oxygenated fluids is restricted and microenvironments of anoxic conditions can prevail (Okland et al., 2012). Generally, open-system oxidation gives way to more restricted circulation as the crust ages. Fractures are clogged with secondary minerals, and access of seawater is restricted through burial of the crust by sediments (Alt, 1995). Seawater compositions evolve with depth and time with the result of less oxidative conditions. Oxygen consumption by Fe^2+^ and other reduced species increase gradually with depth and anoxic conditions can prevail at relative shallow settings (Boettger et al., 2012).

Oxic and anoxic zones can be seen in drill cores as variations in mineralogy, where abundant iron-oxide/oxyhydroxides, ferric micas and smectites represent oxic zones and sulfides (mostly pyrite) and blue–gray–greenish clays like saponite represent anoxic zones. The redox gradients can be steep in subseafloor environments and, thus, contacts between the two zones can be sharp. The presence of anoxic environments in the oceanic crust is further supported by anaerobic isolates from the igneous crust (see section below).

## The Deep Biosphere

Subseafloor basalts are difficult to access and sample compared to seafloor-exposed basalts, and molecular studies from such environments are therefore sparse. The microbiology has been investigated by different approaches including quantitative molecular studies like PCR or FISH, functional gene microarray analysis, and isolation and culturing. There are no direct limitations in these methods for the study of anaerobic compared to aerobic species except for the isolation and cultivation that has to be done under strict anoxic conditions, which is feasible. The presence of representatives of the Phylum Proteobacteria, including the *Alpha-, Beta-*, and *Gammaproteobacteria* lineages (Mason et al., 2010) has been reported from gabbroic layers, as well as anaerobic archaea such as members of the genus *Archaeoglobus* and *Methanosarcina*, both belonging to the phylum Euryarchaeota (Cowen et al., 2003; Orcutt et al., 2010). Lever et al. (2013) reported *Methanosarcinales*, anaerobic methane-oxidizing archaea (ANME-1), and a cluster of microbes that fall within uncultured sulfate reducers. Furthermore, studies of crustal fluids collected on the ocean ridge flanks of the Juan de Fuca Ridge have indicated the presence of members of the phylum Firmicutes, of which some are only distantly related to any known cultivated microorganism and whose mode of life is unclear (Cowen et al., 2003; Nakagawa et al., 2006). Orcutt et al. (2010) showed that the bacterial community of Hole U1301A at the eastern flank of the Juan de Fuca Ridge was dominated by anaerobic members within the phylum Firmicutes. Phylogenetic analysis illustrated close relationship with members of the genus *Ammonifex*, known as thermophilic chemolithoautotrophs capable of growth utilizing hydrogen, formate, or pyruvate coupled to nitrate or sulfate reduction (Orcutt et al., 2010). Also bacteria belonging to the genera *Desulfonatronum* and *Desulfonatronovibrio*, sulfate-reducing bacteria using hydrogen and formate as electron sources, were observed. Thus, a majority of the archaea and bacteria sequenced so far from the igneous crust are anaerobic and/or hydrogen consumers as the methanogens or sulfate reducing bacteria.

Eukaryotes, including fungi, have been neglected in metagenomic studies of the deep biosphere focusing on prokaryotes, but awareness of the presence of eukaryotes in these environments is growing. A few studies indicate that fungi are present in deep-sea sediments, on the seafloor, and in association with hydrothermal vents (Damare et al., 2006; Connell et al., 2009; Le Calvez et al., 2009). Anaerobic fungi have been identified at the Lost City (López-García et al., 2007), in deep-sea sediments (Damare et al., 2006; Nagano and Nagahama, 2012), and may be more abundant in such environments than was previously believed.

In the underlying igneous crust only one fungal isolate from a basalt drill core is reported so far (Hirayama et al., 2015). The isolate was affiliated to the genus *Exophiala* of the order Chaetothyriales and sampled from the North Pond, Mid-Atlantic Ridge. This fungus was isolated and cultured successfully on agar plates with standard fungal medium and artificial seawater. DNA was later extracted and DNA fragments including 18S, 5.8S, 28S rRNA were amplified by PCR. The obtained sequence was compared against the DNA database using BLAST and analyzed to construct a phylogenetic tree. However, little is known of its physiology and metabolic products so far. In future studies it is recommended to sample fungi directly in anoxic microcosms with subsequent aerobic and anaerobic incubation to discern facultative and obligate anaerobic species.

**FIGURE 1:**
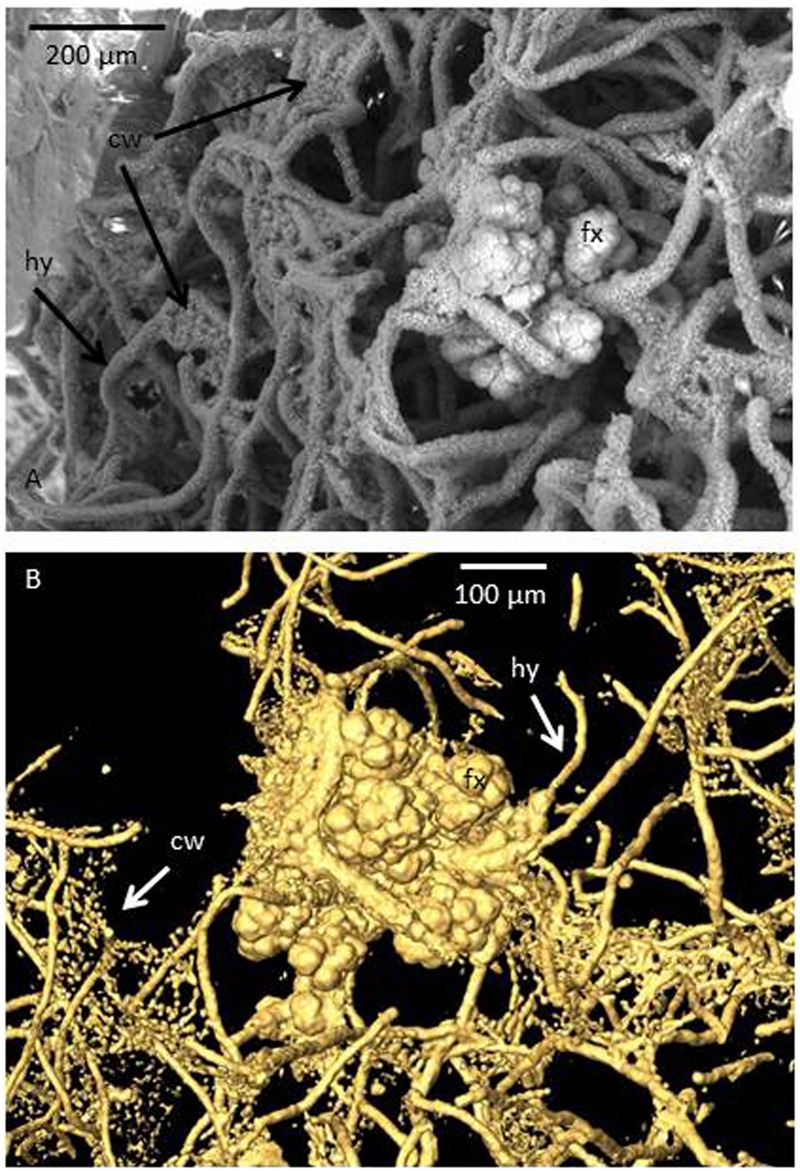
**Images showing the symbiotic relationship between fungi and chemoautotrophic prokaryotes in subseafloor basalts.** The sample was collected at the Koko Seamount in the Pacific Ocean during Ocean Drilling Program 197, and represents a depth of 67.5 m below seafloor and an age of 48 Ma. **(A)** Environmental scanning electron microscopy (ESEM) micrograph of a fossilized fungal mycelium in a basalt vesicle. The fungal hyphae (hy) act as the framework on which prokaryotes have grown. The cauliflower-like structure represents *Frutexites* (fx), a microstromatolitic structure formed by iron oxidizing bacteria. In between hyphae strings of minute cells are attached in a “cobweb-like” (cw) fashion. In the ESEM image these are covered by clay and only seen as sheets compared to **(B)**, a tomographic reconstruction produced by synchrotron-based X-ray tomographic microscopy (SRXTM) in which the sheets become transparent and the cells organized as strings become visual. These were also interpreted as chemoautotropic prokaryotes involved in iron oxidation due to their high iron content. Thus, three types of microorganisms; one fungus and two types of prokaryotes once lived in this consortium in a close spatial interplay (Bengtson et al., 2014).

Several reports of fossilized fungi have recognized the oceanic igneous crust as a thriving fungal habitat (Schumann et al., 2004; Ivarsson et al., 2012, 2013, 2016; Bengtson et al., 2014). Fungal communities have been found in the Pacific Ocean, the Atlantic Ocean, and the Greenland basin, ranging in depths from the top portion of the crust down to ∼950 mbsf (Ivarsson et al., 2011, 2012, 2015a,b,c). Fungi appear to play an important ecological role in these environments as they occur in symbiotic relationships with chemoautotrophic prokaryotes (**Figure 1**) (Bengtson et al., 2014), decompose organic matter introduced by sediments (Ivarsson et al., 2015c), weather minerals like carbonates (Bengtson et al., 2014) and zeolites (Ivarsson et al., 2015a), and are involved in the cycling of Mn (Ivarsson et al., 2015b). Thus, fungi are abundant in the igneous crust and seem to be a substantial geobiological agent.

## Biologically Produced Molecular Hydrogen

The formation of molecular hydrogen in the oceanic crust has been assumed to be the result of abiogenic processes such as: reactions between dissolved gases in the magma; decomposition of methane to graphite (C) and molecular hydrogen (H_2_); radiolysis of water by radioactive uranium, thorium, and potassium isotopes; cataclasis of silicates under stress in the presence of water; and hydrous alteration of ferrous minerals. Hydrous alteration of ultramafic rocks (serpentinization) produces significant quantities of bioavailable molecular hydrogen that theoretically could support a deep biosphere (Nealson et al., 2005; McCollom, 2007; Neubeck et al., 2011, 2014). Aqueous alteration of basalt (palagonitization) has also been shown, in well-circulated ridge flank settings, to produce significant amounts of molecular hydrogen (Türke et al., 2015). Giving that the molecular hydrogen is not swept away by rapid fluid flow it could accumulate to levels high enough to support chemolithoautotrophic life (Türke et al., 2015). However, basalt hosted systems in general produce much lower levels of molecular hydrogen compared to ultramafic hosted systems (McCollom, 2007), and hydrous alteration of ferrous minerals (i.e., magnetite, olivine, and pyroxene) is dependent on fluctuant geochemical constraints like pH, temperature, and a constant access to reduced iron-minerals. Thus, the abiotic production of molecular hydrogen is probably insufficient to ensure a steady supply and support a hydrogen-driven ecosystem over geological periods (Anderson et al., 1998).

An additional source of molecular hydrogen has been proposed by Parkes et al. (2011), who points to biogenic mechanisms for ensuring a steady supply of molecular hydrogen. In sediment slurry experiments (0–100°C) with quartz and basalt minerals, significant formation of molecular hydrogen was observed only in the presence of prokaryotes. A mix of both *Bacteria* and *Archaea* facilitated production of molecular hydrogen by biomechanochemistry in the presence of basalt minerals, and out-competed the abiotic production of molecular hydrogen (Parkes et al., 2011). In the biomechanochemical model the production of molecular hydrogen is stimulated and enhanced by the presence of prokaryotes but not necessarily metabolically produced. An even more productive and continuous source of hydrogen than the biomechanochemical model suggested by Parkes et al. (2011) would be a direct biological production. Molecular hydrogen formed directly from microbiological metabolism, without abiotic involvement, would secure a steady supply of hydrogen as long as the microbiological community is active.

## Anaerobic Fungi

Anaerobic fungi are best known from rumens of herbivores where they are key players in the degradation of lignocellulosic plant fiber (Khejornsart and Wanapat, 2010; Liggenstoffer et al., 2010; Gruninger et al., 2014). Anaerobic fungal species have no mitochondria and are unable to produce energy by either aerobic or anaerobic respiration (Yarlett et al., 1986; O’Fallon et al., 1991). Instead, they meet their energy needs by the fermentation of carbohydrates (general formula C_x_H_2y_O_y_), a process in which the energy source acts as both the electron acceptor and the electron donor. Instead of mitochondria, anaerobic fungi have hydrogenosomes, organelles capable of coupling the metabolism of glucose to cellular energy production. Hydrogenosomes contain hydrogenase, and in the process of decomposing lignocellulosic plant fiber they produce molecular hydrogen, carbon dioxide, acetate, formate, lactate, and ethanol as metabolic waste products (Yarlett et al., 1986; Brul and Stumm, 1994; Khejornsart and Wanapat, 2010; Gruninger et al., 2014).

In the rumen anaerobic fungi exist in relationship with methanogenic archaea, microorganisms that produce methane using carbon dioxide, acetate, or one-carbon substrates as carbon sources and electron acceptors while hydrogen but also acetate, formate, and simple alcohols and methylamines function as an electron donor (Mountfort et al., 1982; Hook et al., 2010; Gruninger et al., 2014). The methanogens increase the enzymatic activity in the fungi through removal of hydrogen, which is followed by a shift in the metabolic activity within the fungi toward a production of methanogenic precursors (Khejornsart and Wanapat, 2010). Experimental studies on cellulose degradation have shown that co-cultures between methanogens and fungi increased the rate of cellulose breakdown dramatically if compared to single-fungus cultures (Bauchop and Mountfort, 1981; Mountfort et al., 1982).

## Anaerobic Microbial Consortia

**FIGURE 2:**
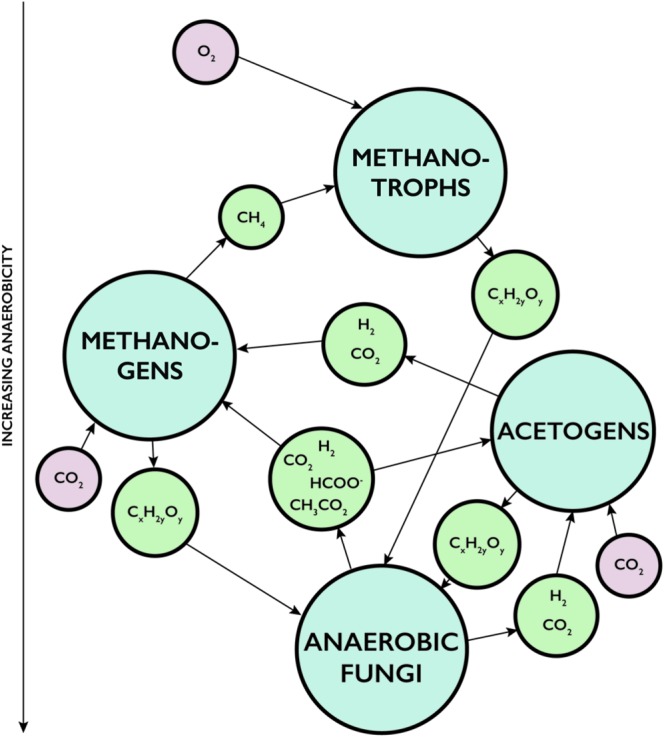
**Scheme for proposed interactions between anaerobic fungi and hydrogen-driven prokaryotic communities such as methanogens and acetogens in an anoxic environment.** Also their impact on associated aerobic microbial communities like methanotrophs. Arrows show metabolic products and reaction pathways. (C_x_H_2y_O_y_) is carbohydrates, (HCOO) is acetate, and (CH_3_CO_2_) is formate.

The aptitude of fungi to enter into symbiotic relationships with chemoautotrophic prokaryotes (**Figure 1**) (Bengtson et al., 2014; Ivarsson et al., 2015a) in deep igneous oceanic crust present a fungal strategy to survive in these rather extreme environments, and it also opens up for a plethora of various community structures, symbiotic partnerships, and metabolic pathways. The geochemical conditions in the subseafloor basalts vary locally on a micro-scale, which influences the microbial composition of the communities. Anoxic environments are inhabited by anaerobic prokaryotes but also likely by anaerobic fungi. In fact, the metabolic waste products of anaerobic fungi – molecular hydrogen, carbon dioxide, acetate, and formate – could serve as energy sources for anaerobic prokaryotes and stimulate their growth (**Figure 2**). Methanogens use carbon dioxide, acetate, or one-carbon substrates as carbon sources and electron acceptors while hydrogen and acetate function as electron donors (Mountfort et al., 1982; Hook et al., 2010). Also formate is consumed by methanogens (Theodorou et al., 1996). Acetogens are another group of anaerobic prokaryotes that would gain from the metabolism of anaerobic fungi. Acetogens can use hydrogen and carbon dioxide to form acetate. But there are also other hydrogen consuming groups identified within the deep-sea oceanic crust that could be favored by the fungal production of molecular hydrogen, such as strict hydrogenotrophs, nitrate reducers, iron reducers, and sulfate reducers (Engelen et al., 2008; Orcutt et al., 2010; Steinsbu et al., 2010; Boettger et al., 2012). An enhanced growth of these communities would result in an increased biomass, which indirectly means an increased pool of available carbohydrates; the base of anaerobic fungal fermentation and metabolism. Fungal decomposition and fermentation of dead prokaryotic cells would trigger further fungal growth. It is also possible that metabolic products of the prokaryotes, such as acetate, could work as accessible carbon sources for the fungal metabolism (Strijbis and Distel, 2010; Wei et al., 2013). Furthermore, prokaryotic consumption of molecular hydrogen increases the enzymatic activity in the fungi, which is followed by a shift in the fungal metabolic activity and enhances the production of molecular hydrogen and acetate available for the prokaryotic portion of the consortia (Khejornsart and Wanapat, 2010). Thus, there would be numerous feedback mechanisms that enable such anaerobic communities of both fungi and prokaryotes to grow and sustain (**Figure 2**). Such a consortium could facilitate indigenous populations independent of the surface biosphere (Stevens and McKinley, 1995) and of abiotically formed hydrogen (Parkes et al., 2011). Some acetogens have also been shown to have, at certain conditions, the ability to oxidize acetate and form hydrogen and carbon dioxide or formate, which then would act as another source of methanogenic substrates (Westerholm et al., 2011).

There are also other feedback mechanisms that would promote microbial growth from this system. Methane produced by methanogens would support the growth of methanotrophic communities that in turn serve as biomass accessible for heterotrophic, including fungal, decomposition, and fermentation. Methanotrophs are both anaerobic and aerobic, but methane is highly volatile and can easily migrate from anoxic to oxic areas of the oceanic crust. Thus, the products of an anaerobic fungal/hydrogen-consuming consortium would spill over to other provinces, including oxic ones, and stimulate microbial growth not directly associated with the consortia.

## Concluding Remarks

We suggest anaerobic fungi as a biological source of molecular hydrogen in anoxic provinces of the igneous oceanic crust. This source might out-compete the abiotic production of hydrogen, at least in basalt hosted systems, where they could represent a steady supply of hydrogen as a base for anaerobic hydrogen-consuming microbial communities such as, i.e., acetogens and methanotrophs. A consortium of anaerobic fungi and prokaryotes could facilitate indigenous populations more or less independent of the surface biosphere (Stevens and McKinley, 1995), and abiotically formed hydrogen (Parkes et al., 2011). The enhanced growth of prokaryotes would represent an increased biomass available for the fungi to feed on and there would be a feedback loop in this system. Decomposition and fermentation of dead microbial communities would promote further fungal growth. There should also be a shift in the fungal metabolic activity toward production of methanogenic precursors. Hydrogen-driven communities can also provide the base for further microbial activities outside the anaerobic realm, such as methanotrophy.

Even though the model is partly hypothetical we wish to stimulate further exploration of the deep biosphere of the igneous oceanic crust including extensive phylogenetic studies of the microbial communities with special emphasis on fungi. Also, large portions of the deep-sea sediments are anoxic, and anaerobic fungi are likely to play an important ecological role there as well.

## Author Contributions

MI designed the study and wrote the paper. AS and SB contributed scientifically to the hypotheses. AN contributed scientifically and designed the illustration.

## Conflict of Interest Statement

The authors declare that the research was conducted in the absence of any commercial or financial relationships that could be construed as a potential conflict of interest.
